# Analysis of microbial communities in wheat, alfalfa, and oat crops after *Tilletia laevis* Kühn infection

**DOI:** 10.3389/fmicb.2024.1343946

**Published:** 2024-08-05

**Authors:** Yuyang Shen, Chen Delai, Taiguo Liu, Wanquan Chen, Guangkuo Li, Haifeng Gao, Li Gao

**Affiliations:** ^1^Key Laboratory of Integrated Pest Management on Crop in Northwestern Oasis, Institute of Plant Protection, Xinjiang Academy of Agricultural Sciences, Ministry of P. R. China, Xinjiang, China; ^2^State Key Laboratory for Biology of Plant Disease and Insect Pests, Institute of Plant Protection, Chinese Academy of Agricultural Sciences, Beijing, China; ^3^College of Plant Protection, Gansu Agricultural University, Lanzhou, China

**Keywords:** fungal community, bacterial community, *Tilletia laevis*, RNA sequencing, soil properties, enzyme activities

## Abstract

Common bunt caused by *Tilletia laevis* Kühn is one of the most serious fungal diseases of wheat. The root–microbial associations play key roles in protecting plants against biotic and abiotic factors. Managing these associations offers a platform for improving the sustainability and efficiency of agriculture production. Here, by using high throughput sequencing, we aimed to identify the bacterial and fungal associations in wheat, alfalfa, and oat crops cultivated in different years in the Gansu province of China. Soil samples (0–6 cm below the surface) from infected wheat by *T. laevis* had significantly more bacterial and fungal richness than control samples as per the Chao1 analysis. We found some dominant fungi and bacterial phyla in infected wheat by *T. laevis*, such as Proteobacteria, Acidobacteria, Actinobacteria, Chloroflexi, Ascomycota, Basidiomycota, and *Mortierello mycota*. We also analyzed the chemical and enzymatic properties of soil samples after *T. laevis* inoculation. The total nitrogen, total kalium (TK), ammonium nitrogen, available kalium, organic carbon, invertase, phosphatase, and catalase were more in *T. laevis*-infected samples as compared to the control samples, while pH, total phosphorus, nitrate nitrogen, available phosphorus, and urease were more in control samples compared to *T. laevis*-infected samples. The results of this study will contribute to the control of wheat common bunt by candidate antagonistic microorganisms and adverse properties of soil.

## 1 Introduction

*Tilletia laevis* Kühn is a threatening pathogen of wheat crops, which causes huge damage worldwide, and mostly sporulation occurs in the plant ovary with host tissues in the kernel slowly replaced by masses of black teliospores (Nguyen et al., 2019). Losses in wheat crops reached 75–80% in many wheat-growing areas of the world (Qin et al., 2020). The relationship between plant pathogens and soil microbes can be either commensalistic, symbiotic, antagonistic, or parasitic (Ruby, 2008; Haegeman et al., 2009). For Tilletia, the characterization of the microbial communities in wheat tissues and rhizosphere soil (Din et al., 2021; Xu et al., 2021), characterization of rhizosphere microbial communities for disease incidence and optimized concentration of difenoconazole fungicide for controlling of wheat dwarf bunt (Jia et al., 2022), and microbiome signature of endophytes in wheat seed response to wheat dwarf bunt caused by *Tilletia controversa* Kühn were explored (Ren et al., 2020). The *T. laevis*, with a fishy smell, leads to the decreased quality and quantity of wheat crops (Lu et al., 2005). Some plants can alter soil biochemical properties (Hussain et al., 2011; Tang et al., 2015), and some plant pathogens can change rhizosphere microbial communities (Zhou and Wu, 2012; She et al., 2017) and alter the relative abundance of other soil-borne pathogens (Din et al., 2021). Soil microbial diversity is not only important for the soil life but also important for soil nutrient cycling (Berendsen et al., 2012). This microbial diversity plays an important role in the health of plants, increasing the soil fertility, and cycling of N, C, and many other nutrients (Berendsen et al., 2012; Miransari, 2013). Previous studies revealed that plant pathogens, such as *T. laevis* (Din et al., 2021), root know nematode (Zhou et al., 2019), and *Erwinia* spp. (She et al., 2017), changed rhizosphere microbial communities. Similarly Mendes et al. (2013) demonstrated that rhizosphere soil microbiome can alter the composition and structure of plant pathogenic and beneficial microorganisms (Mendes et al., 2013). Several studies have shown that soil microbial diversity is influenced by plant pathogens (Zhou et al., 2019; Din et al., 2021). Additionally, environmental factors, like pH, influence soil microbial diversity (Kim et al., 2016; Zhang et al., 2016). Therefore, it is very important to analyze the relationship between environmental factors and soil microbial diversity. However, limited studies have concerns about fungal and bacterial communities under different conditions with different plants after pathogen infection.

Wheat crops is a staple food crop in many countries of the world. Owing to its high amino acid contents, high protein, deliciousness, and use in many products, wheat is used as a food crop throughout the world (Chen et al., 2021). Alfalfa (*Medicago sativa* L.) is a Fabaceae perennial herb and is an important legume crop used for forage worldwide. Moreover, alfalfa is a key source of pollen and nectar throughout the world (Taha, 2015). Oats (*Avena sativa* L.) is an important crop for their high content of functional substances such as phytochemicals, dietary fibers, and several other substances with high nutritional value (Havrlentová et al., 2020). Because of the plant pathogens, the soil microbial community is influenced, which may increase or decrease the relative abundance of soil microorganisms (Din et al., 2021). Additionally, long-term continuous cropping alters the soil microbial community by increasing the relative abundance of soil-borne pathogens in the soil (Yang et al., 2012; Liu et al., 2014; Tang et al., 2015; She et al., 2017). Therefore, there is a need to investigate the effect of a plant pathogen on microbial diversity in different crops that have been growing continuously for a long period. It is known that plant species or soil environment influence the soil microbial diversity composition (Harrison and Bardgett, 2010; Huang et al., 2014). The plants adapt to biotic stresses by modifying the chemistry of their root exudates to assemble a health-promoting microbiome, such as the “cry for help” hypothesis, which provides a mechanistic explanation for previously described soil feedback responses to plant diseases, such as the development of disease-suppressive soils following continuous cultivation of take all-infected wheat (Rolfe et al., 2019). Din et al. (2021) revealed that the diversity and composition of the rhizosphere microbiome associated with wheat crops changed after *T. laevis* infection. However, issues associated with wheat, alfalfa, and oat crops are caused by alterations in the rhizosphere in response to diseases, the cultivation area of these crops has decreased sharply in recent years in Gansu province.

Here, to obtain an inclusive understanding of the rhizosphere soil microorganisms in wheat, alfalfa, and oat crops in Gansu province, China, after *T. laevis* infection, for comparatively exploring fungal and bacterial communities, we subjected fungal and bacterial communities from wheat, alfalfa, and oat cropping fields in Gansu province, China, to high-throughput sequencing, and we used redundancy analysis (RDA) to analyze relationships between soil microbial communities and soil properties with enzyme activities.

## 2 Results

### 2.1 Changes in physical and chemical properties of soil and its enzyme activities by *Tilletia laevis*

The basic chemical characteristics of soil from Gansu Province from the fields of wheat, alfalfa, and oat are listed in Table 1. The total nitrogen (TN), total phosphorus (TP), and total kalium (TK) of soil range from 0.46 to 1.28 g/kg, 0.66 to 0.94 g/kg, and 17.48 to 18.36 g/kg, respectively. Similarly, nitrate nitrogen (NO_3_(−)–N), ammonium nitrogen (NH_4_(+)–N), and available phosphorus (AP) ranged from 0.74 to 54.48 mg/kg, 0.47 to 2.52 mg/kg, and 6.79 to 22.44 mg/kg, respectively. The range of available kalium (AK), organic carbon (OC), moisture content (MC), and pH varied from 109.40 to 352.20 mg/kg, 4.53 to 11.18 g/kg, 0.77 to 15.30%, and 8.25 to 8.80, respectively (Table 1). The activities of various enzymes were investigated in topsoil and rhizosphere soil in various crops. The statistical analysis showed that invertase, phosphatase, urease, and catalase enzymes were significant in different treatments. The invertase was the highest in TFL2, with 46.29 (mg/g), and the lowest in CK1, with 9.19 (mg/g). The phosphatase and urease were the highest in TFL2 and CK4, with 3.29 (mg/g) and 2.01 (mg/g), respectively, and the lowest in CK and TFL1, with 1.32 (mg/g) and 0.14 (mg/g), respectively. Similarly, catalase was the highest in TFL2, with 1.53 (ml/g), and the lowest in CK5, with 1.57 mL/g (Table 2).

**Table 1:** Summary of soil physical and chemical properties under different planting modes.

Treatments	TN (g/kg)	TP (g/kg)	TK (g/kg)	NO_3_(−)-N (mg/kg)	NH_4_(+)-N (mg/kg)	AP (mg/kg)	AK (mg/kg)	OC (g/kg)	MC (%)	pH
CK	0.69 ± 0.008 d	0.94 ± 0.007 i	18.35 ± 0.233 f	30.87 ± 0.483 i	0.98 ± 0.043 e	23.07 ± 0.543 j	184.20 ± 0.837 f	6.22 ± 0.024 d	12.70 ± 0.001 g	8.47 ± 0.005 cd
TFL	0.95 ± 0.005 g	0.82 ± 0.006 h	18.36 ± 0.114 f	28.48 ± 0.218 h	0.81 ± 0.038 d	17.43 ± 0.340 h	186.80 ± 0.837 g	7.42 ± 0.063 f	15.30 ± 0.001 i	8.48 ± 0.007 d
CK1	0.46 ± 0.005 a	0.66 ± 0.012 a	17.56 ± 0.319 ab	0.74 ± 0.061 a	0.47 ± 0.099 a	9.50 ± 0.385 c	109.40 ± 1.140 b	4.53 ± 0.043 a	11.23 ± 0.002 d	8.75 ± 0.019 h
TFL1	0.65 ± 0.009 c	0.67 ± 0.007 ab	17.48 ± 0.155 a	10.71 ± 0.039 d	0.72 ± 0.044 c	6.79 ± 0.140 a	96.60 ± 0.548 a	5.58 ± 0.094 c	12.39 ± 0.002 f	8.62 ± 0.009 f
CK2	0.47 ± 0.005 b	0.67 ± 0.013 b	17.83 ± 0.188bcd	0.99 ± 0.063 a	0.92 ± 0.026 e	11.92 ± 0.329 e	115.60 ± 0.548 c	4.59 ± 0.026 a	12.11 ± 0.001 e	8.80 ± 0.011 i
TFL2	1.28 ± 0.008 j	0.75 ± 0.006 e	18.23 ± 0.251 ef	54.48 ± 0.702 j	1.17 ± 0.039 f	15.56 ± 0.158 f	352.20 ± 2.588 k	11.18 ± 0.101 k	23.16 ± 0.002 j	8.46 ± 0.022 c
CK3	0.96 ± 0.009 g	0.72 ± 0.010 d	17.59 ± 0.158 ab	19.60 ± 0.331 g	1.12 ± 0.024 f	10.90 ± 0.279 d	215.60 ± 1.140 h	8.23 ± 0.106 h	11.20 ± 0.002 c	8.62 ± 0.011 f
TFL3	1.00 ± 0.006 h	0.80 ± 0.006 g	17.82 ± 0.116 bcd	7.67 ± 0.120 c	0.56 ± 0.035 b	17.00 ± 0.398 g	217.40 ± 1.817 i	9.54 ± 0.111 i	0.77 ± 0.002 a	8.62 ± 0.011 f
CK4	0.75 ± 0.015 f	0.72 ± 0.010 d	17.67 ± 0.311 abc	12.75 ± 0.228 e	2.52 ± 0.015 j	6.81 ± 0.145 a	159.00 ± 1.871 e	7.60 ± 0.088 e	12.39 ± 0.002 f	8.25 ± 0.012 a
TFL4	0.71 ± 0.008 e	0.69 ± 0.009 c	17.92 ± 0.294 cde	19.18 ± 0.375 f	1.99 ± 0.168 I	8.14 ± 0.092 b	110.20 ± 0.447 b	6.53 ± 0.064 b	14.75 ± 0.001 h	8.35 ± 0.008 b
CK5	0.47 ± 0.009 b	0.81 ± 0.010 g	17.63 ± 0.315 abc	0.79 ± 0.012 a	1.63 ± 0.078 g	22.44 ± 0.261 i	117.60 ± 0.548 d	4.76 ± 0.046 d	10.22 ± 0.001 b	8.71 ± 0.010 g
TFL5	1.07 ± 0.007 i	0.77 ± 0.009 f	18.10 ± 0.118 def	5.49 ± 0.130 b	1.78 ± 0.079 h	11.21 ± 0.193 d	240.80 ± 1.483 j	9.90 ± 0.077 j	15.30 ± 0.000 i	8.58 ± 0.005 e

Different letters indicate significant differences between different planting modes (ANOVA, *p* < 0.05) analysis.

Note: total nitrogen (TN), total phosphorus (TP), total kalium (TK), nitrate nitrogen (NO3(−)–N), ammonium nitrogen (NH4(+)–N), available phosphorus (AP), available kalium (AK), organic carbon (OC), moisture content (MC).

**Table 2:** Summary of soil enzyme activities under different planting patterns.

Treatments	Inverting (INV) (mg/g)	Phosphatase (PHO) (mg/g)	Urease (URE) (mg/g)	Catalase (CAT) (ml/g)
CK	13.07 ± 0.125 b	1.32 ± 0.068 a	1.70 ± 0.028 h	1.61 ± 0.035 b
TFL	21.63 ± 0.162 f	1.75 ± 0.020 c	1.90 ± 0.077 i	1.98 ± 0.009 f
CK1	9.19 ± 0.120 a	1.58 ± 0.080 b	0.17 ± 0.017 a	1.73 ± 0.062 d
TFL1	17.82 ± 0.340 e	2.23 ± 0.069 e	0.14 ± 0.014 a	1.85 ± 0.039 e
CK2	14.22 ± 0.192 c	1.85 ± 0.064 cd	0.32 ± 0.027 b	1.75 ± 0.027 d
TFL2	46.29 ± 0.898 L	3.29 ± 0.055 h	0.73 ± 0.031 c	1.53 ± 0.016 a
CK3	38.29 ± 0.206 k	2.98 ± 0.040 g	1.15 ± 0.026 f	1.95 ± 0.046 f
TFL3	24.30 ± 0.722 g	1.97 ± 0.192 d	0.80 ± 0.020 d	1.88 ± 0.056 e
CK4	34.02 ± 1.112 i	3.10 ± 0.208 g	2.01 ± 0.091 j	1.88 ± 0.035 e
TFL4	27.83 ± 0.565 h	2.69 ± 0.135 f	0.16 ± 0.017 a	1.66 ± 0.023 c
CK5	15.61 ± 0.336 d	1.39 ± 0.063 a	1.06 ± 0.041 e	1.57 ± 0.026 ab
TFL5	35.84 ± 0.503 j	2.58 ± 0.109 f	1.53 ± 0.043 g	1.96 ± 0.019 f

Different letters indicate significant differences between different planting modes (ANOVA, *p* < 0.05) analysis.

### 2.2 The influence of different types of soil on microbial diversity by *Tilletia laevis*

Across all (Garrett et al., 2018) rhizosphere soil samples, a total of 4,551,828 original bacterial sequences were obtained and 4,415,176 high-quality bacterial sequences were obtained from all samples. Similarly, 4,178,521 original fungal sequences were obtained, of which 3,900,304 were high-quality sequences. These bacterial and fungal sequences were on OTUs with 97% similarity levels. A total of 13,628 bacterial OTUs and 3,606 fungal OTUs were left after leveling (Supplementary Table S1).

### 2.3 Diversity and species richness of bacterial and fungal community by *Tilletia laevis*

**Figure 1:**
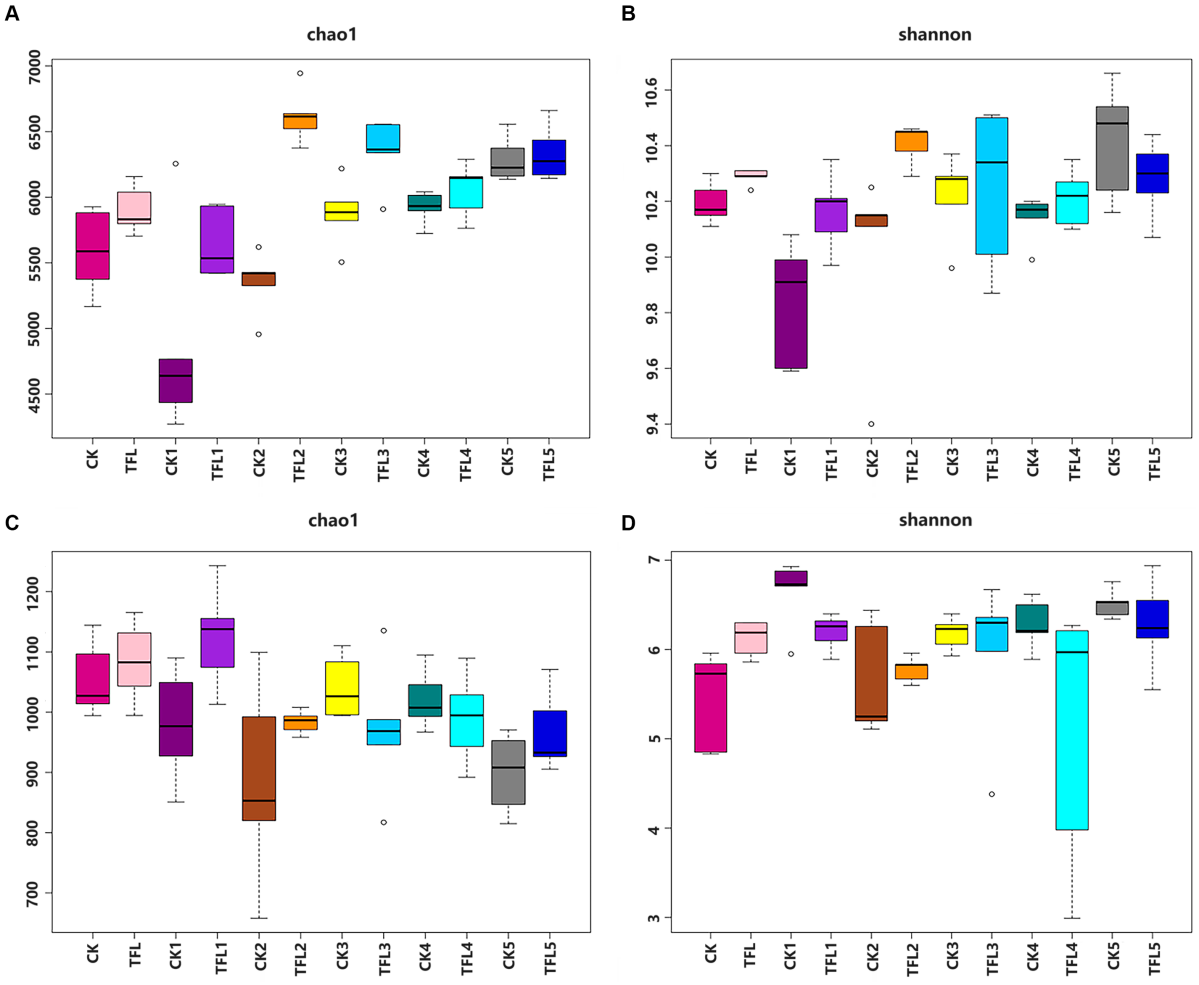
Chao1 and Shannon diversity analysis in the top layer and rhizosphere soil of wheat, alfalfa, and oat crops from pathogen trials inoculated with *T. laevis*. **(A)** Chao1 analysis of bacterial community. **(B)** Shannon analysis of bacterial community. **(C)** Chao1 analysis of fungal community. **(D)** Shannon analysis of fungal community.

**Figure 2:**
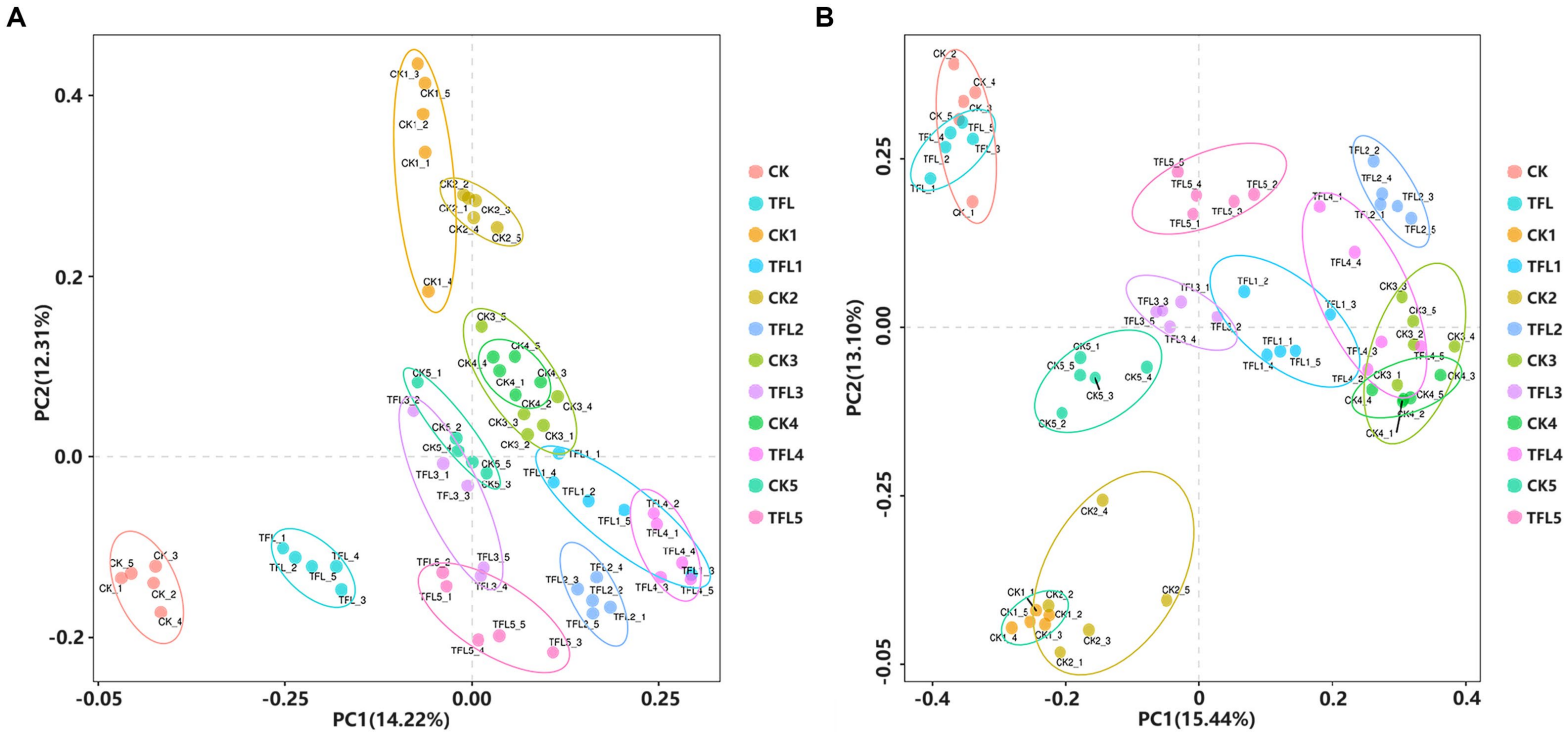
PCA of the OTUs detected major variations in the bacterial and fungal communities in three (wheat, alfalfa, and oat) crops. The OTUs differentiate based on the plant type and soil type. **(A)** PCAs analysis for bacterial community OTUs. **(B)** PCAs analysis for fungal community OTUs.

Alpha diversity was analyzed based on the Chao1 and Shannon diversity indexes to assess the robustness of the dataset (Figure 1). The Chao1 index reflects species richness in samples, without considering the abundance of every species (Qiao et al., 2017). For bacteria, results showed that TFL2 and TFL3 soils have significantly higher species richness compared to CK1-5, TFL1, 4–5, TFL, and CK measured by Chao 1 index (Figure 1A). Additionally, for the Shannon diversity estimates, the CK5, TFL3 and TFL2 soils have significant higher diversity compared to TFL1, 4–5, CK1, 3–4, TFL, and CK (Figure 1B). For fungi, TFL1 and TFL rhizosphere soils have significant higher species richness than CK, CK1-5, and TFL2-5(Figure 1C). Additionally, for the Shannon diversity estimates, CK1 and TFL5 soils have significant higher diversity than CK, TFL, TFL1, 2–4, and CK2-5 (Figure 1D). We further conducted a comparison of the species diversity among different microbial communities. The principal coordinates analysis (PCoA) based on the Bray–Curtis distance between samples was visualized to analyze the differences in bacterial and fungal community diversity between groups. The samples of the same replicates clustered together indicated the level of significance. Additionally, samples formed distinct clusters, revealing that the largest source of variation was noted in the microbial community. The PCoA analysis bacterial OTUs showed the maximum variation of 14.22% (PC1) and 12.31% (PC2), as shown in Figure 2A and fungal OTUs showed the maximum variation of 15.44% (PC1) and 13.1% (PC2), as shown in Figure 2B.

### 2.4 Dominant phyla and genera of bacterial and fungal communities

**Figure 3:**
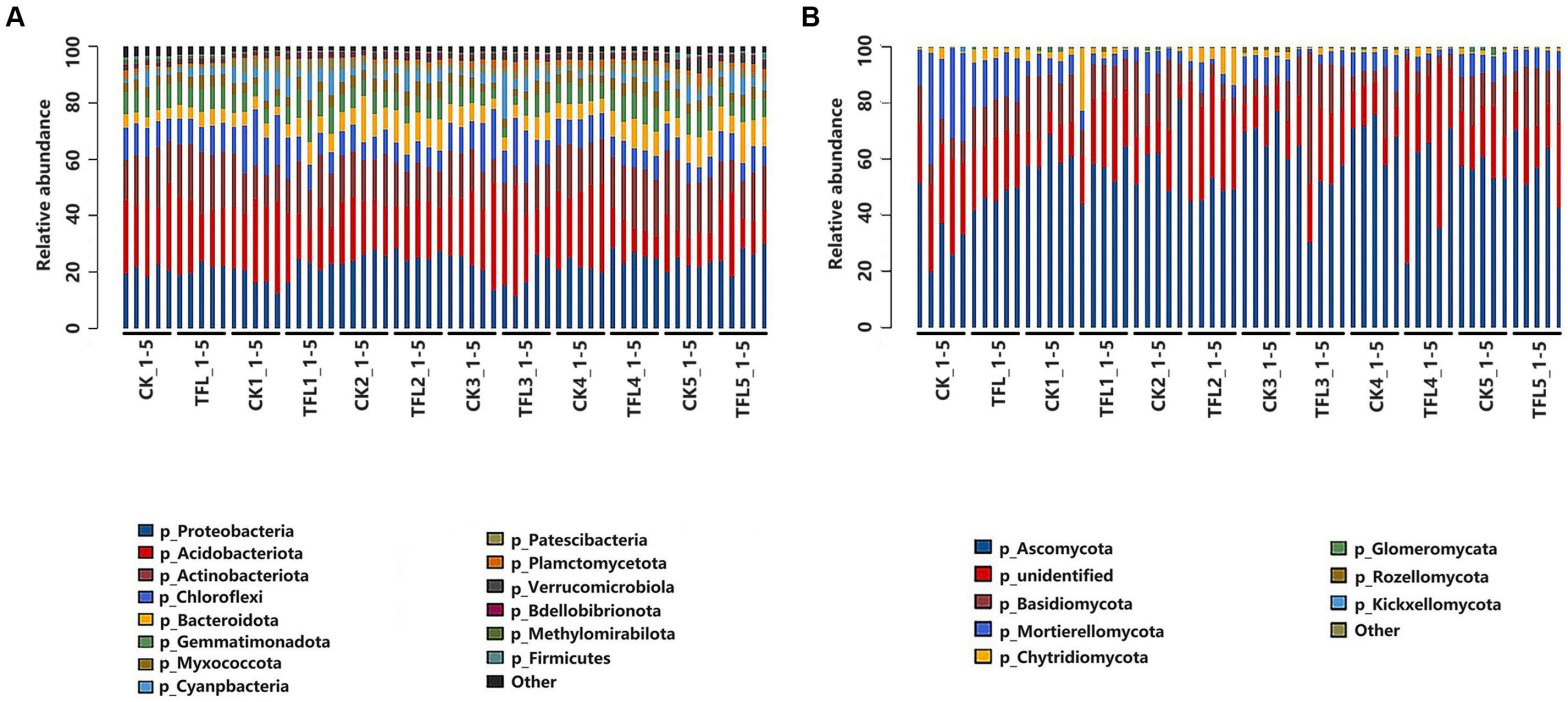
The relative abundance of the dominant bacterial and fungal taxa in Gansu province in three (wheat, alfalfa, and oat) crops at the phylum and genus levels. **(A)** Relative abundance of bacterial community at the phylum level. **(B)** Relative abundance of fungal community at the phylum level. Sequences not classified into any known group were designated as “other”.

There were differences in the diversity indexes within the 12 samples analyzed demonstrating specific trends within different soil samples. The sequences that could not be classified into any known group are allocated as other and unidentified. The relative abundance of bacterial and fungal communities of *T. laevis* infected and control samples were different from each other. For bacteria, a total of 12 were distributed at the phylum level. Results showed that the phylum Proteobacteria, Acidobacteria, Actinobacteria, Chloroflexi, and Gemmatimonadetes were the dominant phyla in above samples than other phylum (Figure 3A). Similarly, for the fungus, the dominant phyla were Ascomycota, Basidiomycota, and Mortierellomycota, as compared to other phyla (Figure 3B).

### 2.5 Correlation between microbial communities with soil properties and enzyme activities

**Figure 4:**
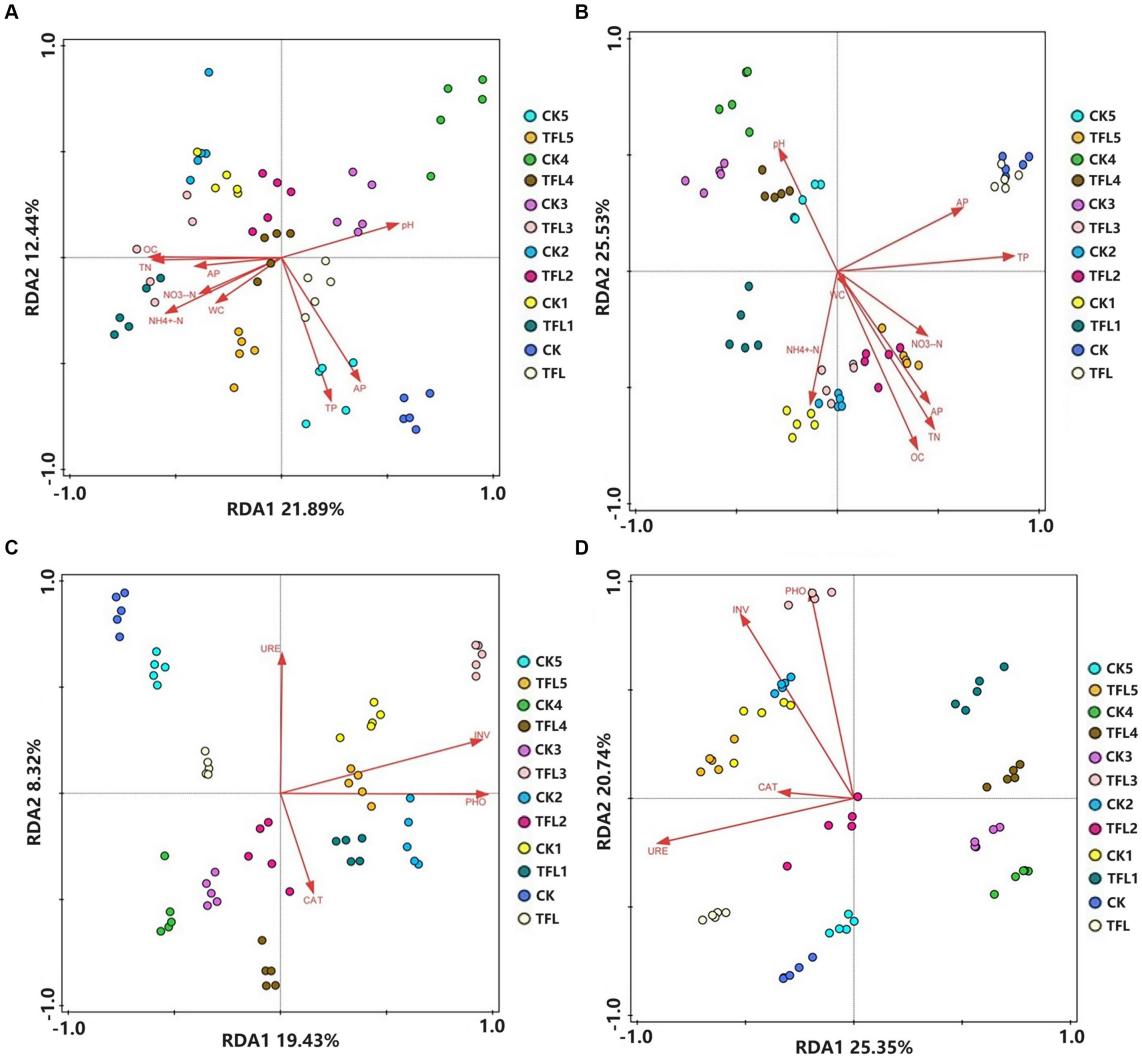
Redundancy analysis (RDA) based on bacterial and fungal OUT data with chemical properties and enzyme activity in three (wheat, alfalfa, and oat) crops after *T. laevis* infection. **(A)** The relationship between bacterial community and chemical properties of soil. **(B)** The relationship between fungal community and chemical properties of soil. **(C)** The relationship between bacterial community and enzyme activity. **(D)** The relationship between fungal community and enzyme activity.

In all samples, the bacterial and fungal OTUs were correlated with soil properties and enzyme activities using redundancy analysis (RDA). The RDA based on OTU reads, soil properties, and enzyme activities were carried out for the various soil samples in Gansu province, China. The relationship between bacterial communities and soil properties is illustrated in Figure 4A (RDA1 = 21.89%, RDA2 = 12.44%), the relationship between fungal communities and soil properties is illustrated in Figure 4B (RDA1 = 29.22%, RDA2 = 25.53%). Similarly, the relationship between bacterial communities and enzyme activity is illustrated in Figure 4C (RDA1 = 19.43%, RDA2 = 8.32%) and relationship between fungal communities and enzyme activity are illustrated in Figure 4D (RDA1 = 25.35% and RDA2 = 20.74%). The length of the arrow in the RDA plot indicates the degree of correlation among sample distribution, soil properties, and enzymatic activity. The results demonstrated that TP, AP, pH, OC, and TN showed the most significant correlation with bacterial community, while AP, NO_3_-H, and WC showed the least correlation with bacterial community structure in all soil samples (Figure 4A). Similarly, organic carbon (OC), TN, TP, AP, pH and NH_4+_-N showed the most significant correlation with fungal community, while NO_3_–N and moisture content (MC) revealed the least correlation with fungal community structure in all soil samples (Figure 4B). Additionally, bacterial community and enzyme activity analysis revealed that URE, INV, and PHO exhibited the most significant correlation in all samples, except CAT, which revealed the least correlation (Figure 4C). Moreover, PHO, INV, and URE enzymes showed the most significant correlation with fungal community structures in all samples (Figure 4D).

## 3 Discussion

In this study, using high-throughput sequencing, we analyzed bacterial and fungal communities in wheat, alfalfa, and oat crop fields in Gansu Province, China. According to the α-diversity analysis, the overall diversity of bacterial and fungal community compositions differed among the soil samples. The Chao1 α-diversity and Shannon analysis revealed that the diversity of microbial communities is different in different crops (Figure 1). This may be due to different soil characters and crops in different periods. For bacteria, the *Proteobacteria*, *Acidobacteria*, *Actinobacteria*, *Chloroflexi*, *Gemmatimonadetes,* and *Bacteroidetes*, while for fungus, the *Ascomycota*, *Basidiomycota,* and *Mortierellomycota* were the dominant phyla (Figure 3), which was by the findings of previous studies (Zhou et al., 2019; Wang et al., 2020; Din et al., 2021). These phyla were also dominant in fields of soybean (Li et al., 2010), peanut (Li et al., 2014), and tobacco (She et al., 2017), as well as in *T. laevis* (Din et al., 2021) and root-knot nematode-infected (15)fields. The members of *Proteobacteria* play an important role in S, N, and C in soil (Nosheen et al., 2016). Previous studies revealed that there is greater abundance of *Proteobacteria* in fertile soil as compared to diseased soil (Wang et al., 2017). However, in our results, the percentage of *Proteobacteria* was the highest in TFL2 (alfalfa rhizosphere soil infected with *T. laevis*) from different crops after *T. laevis* infection. The *Acidobacteria* and *Actinobacteria* are key players in the suppression of fungal pathogen F. oxysporum (Trivedi et al., 2017). The *Bacillus* is a genus of *Firmicutes*, which has the role of controlling soil-borne pathogens and can stimulate plant growth activities as a beneficial microbe (Jos et al., 2008). For instance, *Bacillus* spp. inhibits *R. solanacearum* infection, which causes bacterial wilt (Guo et al., 2004; Tan et al., 2010; Muhae-ud-Din et al., 2018). Additionally, application of *Bacillus* spp. as a fertilizer can increase the soil microbial diversity (Huang et al., 2012). Therefore, *Firmicutes* are the best options to improve the soil microbial community and are influenced by soil-borne pathogens (Wang et al., 2020; Din et al., 2021). In our study, the relative abundance of fungal and bacterial rhizosphere microorganisms significantly changed in *T. laevis*-inoculated samples as compared to control samples with the increased abundance of *Ascomycota*, *Basidiomycota*, *Proteobacteria,* and *Acidobacteria*. These changes could be attributed to a change in the root exudation patterns in the presence of soil-borne pathogens, a higher prevalence of dead roots, and microbial competition (Jones et al., 2009; Berendsen et al., 2012; Zahar et al., 2014; Dudenhöffer et al., 2016; Gu et al., 2016). These rhizosphere soil microorganisms have a role in changing redox conditions, C flow, soil pH, and the production of rhizodeposits, including the release of root exudates of various natures (Hinsinger et al., 2003; Jones et al., 2009; Dennis et al., 2010). In our results, *Ascomycota*, *Basidiomycota,* and *Mortierellomycota* were the dominant phyla, which were consistent with the findings of previous studies (Xu et al., 2018; Din et al., 2021). The *Basidiomycota* and *Ascomycota* are important groups of fungi in most types of soils (Wallenstein et al., 2007; Unterseher et al., 2013), and species of these phyla are involved in crop cycling by degrading organic substances (Unterseher et al., 2013; Purahong et al., 2016). We observed significant changes in the relative abundance of *Basidiomycota* and *Ascomycota* in our samples, especially in CK-4, the relative abundance of *Ascomycota* was the highest compared to other soil samples.

The soil properties, including available N and soil pH, are influenced directly or indirectly by plant pathogens (Lazcano et al., 2021). Soil properties play an important role in plant nutrient acquisition and resistance to biotic and abiotic stresses (Eaton et al., 2012; Yu et al., 2022; Tiecher et al., 2023), such as adequate total nitrogen (TN) levels, vigorous plant growth, and higher yields. Phosphorus (TP) and total kalium deficiency can limit crop growth and yield, and low levels of NO_3_(−)-N can limit plant growth. NH_4_(+)-N can be influenced by soil pH and temperature, Adequate phosphorus (AP) levels are crucial for early root development and flowering. Available kalium (potassium) deficiency can increase susceptibility to diseases and stress. High organic carbon (OC) content, moisture content (MC), and pH can influence nutrient availability, microbial activity, and plant growth, excessive and low levels both will hinder nutrient uptake. Hence, we used RDA analysis for the relationship between environmental factors (including soil T, available K, soil pH, TN, and urease activity) and soil microbial composition. RDA results showed that environmental factors differentially affected the fungal and bacterial communities, which were proven by various previous studies (Zhang et al., 2005; DeAngelis et al., 2015; Zhou et al., 2017). Urease catalyzes the breakdown of urea into NH_3_ and CO_2_, which may be good for soil quality (Jezierska-Tys and Rutkowska, 2014). The plants and rhizosphere soil microorganisms release urease enzymes (Follmer, 2008).

The plant pathogens cause a decline in the urease activity, and positive correlations between soil micro-organisms and urease have been previously found (Lazcano et al., 2021).

Soil microbial communities were altered in response to pathogen infection, leading to changes in soil enzymatic activities and nutrient availability (Mendes et al., 2013). Pathogen-infected plants may exhibit altered nutrient uptake and cycling dynamics. For example, *Phytophthora infestans* infection in potato plants can lead to changes in phosphorus cycling and availability in the soil; pathogen infections can decrease crop yield globally, with significant variation depending on the pathogen and crop species (Garrett et al., 2018). Pathogen infections can alter the composition and function of soil microbial communities, which play crucial roles in nutrient cycling, disease suppression, and plant health. For example, *Fusarium oxysporum* infection in different common beans has been shown to reduce microbial diversity and alter soil bacterial community composition (Hollander, 2018). Similarly, in our results, the urease activity changed after *T. laevis* inoculation in different crops (Table 2). Previous studies revealed that N has a role in regulating the rhizosphere soil microbial community (Cleveland et al., 2007; Högberg et al., 2014), and urease activity increased by the N application from 247 to 433 mg/kg (Liang et al., 2016; Lei et al., 2018). Therefore, N provides a good means to increase the urease activity to increase the soil micro-biota. However, a high concentration of ammonia can reduce the activity of the urease enzyme (Piotrowska and Wilczewski, 2012). Additionally, TN has a major role in influencing the fungal and bacterial community (Wang et al., 2020).

In conclusion, according to the RDA analysis of rhizosphere microorganisms and environmental factors in Gansu province, a positive correlation was noted in the chemical properties and enzyme activity of rhizosphere and top-layer soil. We explored some dominant fungi and bacterial phyla in the rhizosphere and top soil in infected wheat by *T. laevis*, such as Proteobacteria, Acidobacteria, Actinobacteria, Chloroflexi, Ascomycota, Basidiomycota and Mortierellomycota, which were related to *T. laevis*, we may reduce the content of this may contribute to the control of *T. laevis* shortly, and we may isolate these to explore the interaction with *T. laevis* (Jin et al., 2023; Zhou et al., 2023). Even though some taxa belong to the same genus, they can have different functions in the control of different pathogens. Additionally, nitrogen, total kalium, ammonium nitrogen, available kalium, and organic carbon were increased after *T. laevis* infection, so, reducing these elements may also contribute to controlling wheat’s common bunt disease which is caused by *T. laevis*. Hope shortly, we can control the wheat’s common bunt disease with efficient and friendly microbiology and the elements mentioned above.

## 4 Materials and methods

### 4.1 Site description and sample collection

The experimental site was located in Gansu Province, 32°11′ - 42 °57″ N and 92 °13 ′-108 ° 46″ (E). The soil samples were collected from a depth of 6 cm with a stainless-steel cylindrical driller and immediately stored in a portable refrigerator at −20°C for further use. The samples were passed out from a 2 mm sieve to remove the debris and stored at −20°C for next use. We collected samples from topsoil and rhizosphere soil from five plants and pooled them into one sample. A total of 12 soil samples from wheat, alfalfa, and oat crops were collected and stored in plastic bags and shifted on ice to the laboratory. One-half of each soil sample was stored at −20°C for biochemical and biological analyses, and the remaining were used for chemical analysis. Every sample was investigated in triplicate. Detailed information about samples is illustrated in Table 3. The *T. laevis* culture was collected from the Institute of Plant Protection, Chinese Academy of Agricultural Sciences, Beijing, China. With the teliospores from infected wheat tassels and the concentration of *T. laevis,* infectious hyphae were adjusted to 10^6^ cfu/mL with an OD_600_ of 0.15. Five inoculations of *T. laevis* infectious hyphae were inoculated into the root zone of all the above-mentioned crop varieties, with three biological replicates as described (Din et al., 2021), and three sets of each variety were used as controls.

**Table 3:** Samples information.

Number	Soil category	Sample name	Crop name
1	Wheat Rhizosphere soil CK	CK	*Triticum aestivum*
2	Wheat Rhizosphere soil TFL	TFL	
3	1-year-old *M. sativa* field interstitial soil CK	CK1	*Medicago sativa*
4	1-year-old *Medicago sativa* field interstitial soil TFL	TFL1	
5	Rhizosphere soil of 1-year-old *M. sativa* field CK	CK2	
6	Rhizosphere soil of 1-year-old *M. sativa* TFL	TFL3	
7	2-year-old *M. sativa* field interstitial soil CK	CK3	
8	2-year-old *M. sativa* field interstitial soil TFL	TFL3	
9	Rhizosphere soil of 2-year-old *M. sativa* CK	CK4	
10	Rhizosphere soil of 2-year-old *M. sativa* TFL	TFL4	
11	1-year-old *Avena sativa L* rhizosphere soil CK	CK5	*Avena sativa*
12	1-year-old *A. sativa L* rhizosphere soil TFL	TFL5	

### 4.2 Analysis of soil basic properties and enzymatic properties

Soil basic properties, including TP, AP, pH, NO_3_(−)-N, NH_4_(+)-N, OC, and TN were analyzed by using redundancy analysis (RDA) with CANOCO 4.5 (Biometrics, Wageningen, The Netherlands). These basic properties of soil were analyzed by following the method of previous reports (Xu et al., 2018; Wang et al., 2020). The sodium phenate and sodium hypochlorite colorimetric methods were used to determine soil urease and other enzyme activities (Vlek et al., 1980).

### 4.3 DNA extraction and PCR amplification

DNA extraction was performed from 5 gm of each homogenized soil sample as previously described (DeSantis et al., 2005) and purified using the PowerSoil® DNA isolation kit (MO BIO, Carlsbad, CA, United States), according to the manufacturer’s instructions. DNA concentration was quantified on a NanoDrop spectrophotometer (Thermo Scientific). The primer sequences for *T. laevis* were ITS1F (5- CTTGGTCATTTAGAGGAAGTAA −3) and ITS2 (5- TGCGTTCTTCATCGATGC -3). PCR amplification was performed by using 25 μL mixture, including 12.5 μL KAPA 2G robust hot start ready mix, 1 μL forward primer (5 μM), 1 μL reverse primer (5 μM), 5 μL DNA (30 ng), and 5.5 μL ddH_2_O. Following an initial denaturation at 95°C for 5 min, PCR was cycled 28 times at 95°C for 45 s, 55°C for 50 s, and a final extension at 72°C for 10 min. PCR products were purified using the AMPure XP kit (Beckman Coulter, Life Sciences).

### 4.4 High-throughput sequencing and data analysis

Deep sequencing was performed on MiSeq platform allergens Technology Inc. (Biotechnology, Beijing). After the run, image analysis, base calling, and error estimation were performed using Illumina analysis pipeline version 2.6. The samples were sequenced based on the following bases: (1) the sequence with precise primers and bar codes; (2) quality score ˃20; and (3) the sequences >230 bp in length. The data analysis was done by following the method of published procedures (Wang et al., 2020; Din et al., 2021). Additionally, visualization of beta-diversity information was achieved via ordination plotting with non-metric multidimensional scaling (NMDS) (Tian et al., 2018).

## Data availability statement

The datasets presented in this study can be found in online repositories. The names of the repository/repositories and accession number(s) can be found in the article/Supplementary material.

## Author contributions

YS: Data curation, Writing – original draft. CD: Data curation, Writing – original draft. TL: Formal analysis, Writing – review & editing. WC: Formal analysis, Writing – review & editing. HG: Formal analysis, Writing – review & editing. LG: Conceptualization, Data curation, Funding acquisition, Investigation, Resources, Supervision, Writing – original draft, Writing – review & editing. GL: Formal analysis, data curation, Writing – review & editing.
